# Effects of Total Ginsenosides on the Feeding Behavior and Two Enzymes Activities of *Mythimna separata* (Walker) Larvae

**DOI:** 10.1155/2015/451828

**Published:** 2015-05-17

**Authors:** Ai-Hua Zhang, Shi-Qiang Tan, Yan Zhao, Feng-Jie Lei, Lian-Xue Zhang

**Affiliations:** ^1^College of Chinese Medicinal Materials, Jilin Agricultural University, Changchun 130118, China; ^2^Tianjin University of Traditional Chinese Medicine, Tianjin 300193, China

## Abstract

Ginsenosides, the main effective components of *Panax ginseng* C.A. Meyer and *Panax quinquefolius* L., are important allelochemicals of *ginseng*. Although many studies have targeted the pharmacological, chemical, and clinical properties of ginsenosides, little is known about their ecological role in *ginseng* population adaptation and evolution. Pests rarely feed on *ginseng*, and it is not known why. This study investigated the effects of total ginsenosides on feeding behavior and activities of acetylcholinesterase (AChE) and glutathione s-transferase (GST) in *Mythimna separata* (Walker) larvae. The results showed that the total ginsenosides had significant antifeeding activity against *M. separata* larvae, determined by nonselective and selective antifeeding bioassays. In addition, the total ginsenosides had inhibitory effects on the activities of GST and AChE. The antifeeding ratio was the highest at 8 h, then decreased, and was the lowest at 16 h. Both GST and AChE activities decreased from 0 h to 48 h in all total ginsenosides treatments but increased at 72 h. Total ginsenosides had antifeeding activity against *M. separata* larvae and inhibitory effects on the activities of GST and AChE.

## 1. Introduction

In the long process of adaptation to the environment, plants have developed a chemical response to stress called allelopathy, the release of allelochemicals [1, 2]. Terpenoids are a type of allelochemical and play an important role in regulating plant populations and ecological systems [3, 4].

*Ligularia virgaurea* secretes terpenoids that inhibit seed germination of other species [5, 6].* Duranta repens* extracts have been shown to inhibit oviposition, feeding, and development of* Plutella xylostella* [7, 8]. Terpenoids from Meliaceae plants have shown insecticidal action against* Pieris occidentalis* and* Leucania separata* [9–12].

*Ginseng* (*Panax ginseng* C.A. Meyer) is a highly popular and valuable traditional Chinese medicine and tonic. The major* ginseng* producers of the world are China, Korea, Japan, and Russia [13, 14].* Ginseng* can grow for more than one hundred years, but it requires strict ecological conditions that are limited. Wild* ginseng* populations maintain a specific distance internally between plants [15].* Ginseng* has triterpene saponins, and few insects eat its stems or leaves.* Ginseng* triterpene saponins change the soil microbial population structure and inhibit* ginseng* seed and seed germination by other species [13–20].

Ginsenosides belong to the group triterpenes and are becoming a popular topic of research in pharmacology, medicine, and clinical drug development in both China and abroad. However, there is little information as to why* ginseng* synthetizes high levels of ginsenosides (contents of more than 3%) and the significance of ginsenosides for the plant's growth, development, and population dynamics.* Ginseng* contains a wide variety of triterpene saponins (found in more than 60 species), which suggests that there are a large number of metabolic pathways (including highly evolved metabolic pathways). Based on the scarcity of information, this study discusses the effects of total ginsenosides on the feeding behavior and two enzyme activities of* Mythimna separata* (Walker) larvae. The results of this study will enable further understanding of the effect of triterpenes on plant population adaptation and evolution. It is also useful to investigate the mechanisms of Chinese herbal medicines and to add to the reference information for phytochemotaxonomy.

## 2. Materials and Methods

### 2.1. Insects

*Mythimna separata* (Walker) adults were collected at the test base of Jilin Agricultural University in the early summer of 2011. The offspring of these adults were reared on grain sorghum leaves under an LD 16 : 8 h photoperiod at 22 ± 1°C with 70–80% relative humidity and never had contact with insecticides [21].

### 2.2. Chemicals

The total ginsenosides (purity ≥ 80%, UV method) were purchased from Jilin Hongjiu Biotech Co., Ltd. AChE and GST reagent kits were purchased from the Nanjing Jiancheng Bioengineering Institute. A Lowry Protein Assay Kit was purchased from Beijing Dingguo Changsheng Biotech Co., Ltd.

### 2.3. Bioassay for Feeding Behavior

A leaf disc bioassay was used to test* M. separata* larvae [22, 23]. The ginsenoside concentrations were 2.0%, 1.0%, and 0.5% (mass fraction (MF)). These concentrations are within the range of ginsenoside levels normally found in* P. ginseng*. One day prior to the assay, newly molted 4th-instar larvae were placed individually into Petri dishes (9 cm diameter). After starvation for 48 h, 6 excised grain sorghum leaf discs (Φ = 15 mm), soaked in solutions with different concentrations of ginsenosides for 30 min (control discs received distilled water only), were supplied to all larvae. The area of feeding on the leaves was measured every 8 h, and the corresponding antifeeding activity was calculated (nonselective and selective antifeeding ratios). Each test was repeated three times.

### 2.4. Enzymes Activity Tests

The effects of ginsenosides on larvae of * M. separata* larvae were tested at concentrations of 2.0%, 1.0%, and 0.5% (MF). The ginsenosides were dissolved with distilled water. Fresh clean grain sorghum leaves were punched into leaf discs with a puncher, and the leaf disc was soaked in different concentrations of ginsenoside solutions for 30 min and air-dried prior to use. The same volume of distilled water without ginsenosides was used for the control group. Fourth-instar larvae were maintained for 48 h without access to food. Larvae with the same weights were then selected and either were allowed to feed on the leaf discs containing ginsenosides or were placed in the control group. The leaves were replaced with the fresh ones every 2 days because of the possible degradation of the ginsenosides. Homogenates for the two enzyme activities (glutathione s-transferase (GST) and acetylcholinesterase (AChE) activity) of * M. separata* larvae were collected every 24 h. Each test was repeated three times.

Homogenates of the digestive tract of the larvae were prepared as follows. The digestive tract was dissected from individuals of each group and washed with phosphate-buffered saline (pH 7.0) to remove the gut contents. To this, 5 mL of cold PBS was added and the solution was centrifuged for 20 min (4°C, 10000 r/min). The supernatant is the homogenate. The protein content of the supernatant fluid was measured using the Lowry Protein Assay Kit staining method [24, 25] with bovine serum albumin as the standard.

GST activity was assessed according to the method of Jaclyn and Min Lü [26, 27] by measuring GSH conjugation with 1-chloro-2,4-dinitrobenzene (CDNB). CDNB activity changes with the substrate concentration in a linear relationship. A visible-ultraviolet spectrophotometer measured absorption at 412 nm to detect product formation. All reactions took place in 0.1 M potassium phosphate-buffer containing 1 mM GSH and 0.01–3 mM CDNB. GST activity was determined according to the following equation:

(1) $$\begin{array}{c} Y=\frac{{\text{OD}}_{\alpha }-{\text{OD}}_{\beta }}{{\text{OD}}_{\delta }-{\text{OD}}_{\varepsilon }}\times C_{\sigma }\times N÷T*\left(A\times C_{\text{prot}}\right), \end{array}$$

where *Y* represents GST activity (*μ*mol/min/mg prot), OD_*α*_ represents the absorbance of the control group, OD_*β*_ represents the absorbance of the experiment group, OD_*δ*_ represents the absorbance of the reference group, OD_*ε*_ represents the absorbance of the blank group, *C*_*σ*_ represents the standard concentration (20 mM), *N* is the dilution factor of the reaction system (6),  *T* is reaction time (10 min), *A* is the sample volume (mL), and *C*_prot_ represents the protein concentration of the sample.

AChE activity was determined using the method of Ellman et al. [28]. The enzyme activity was measured by assessing the increase of yellow color produced from thiocholine when it reacts with the dithiobisnitrobenzoate ion. It is based on the coupling of the following reactions:

(2) $$\begin{array}{c} \text{acetylthiochline}\overset{\text{enzyme}}{\to }\text{thiocholine}+\text{acetate}\\ \text{thiocholine}+\text{dithiobisnitrobenzoate}⟶\text{yellow}\;\;\text{color} \end{array}$$

The AChE activity was spectrophotometrically measured at 412 nm (UV-754, Shandonggaomi, UV visible-ultraviolet spectrophotometer). The enzyme activities were expressed as *μ*mol/min/mg protein. AChE activity was determined according to the following equation:

(3) $$\begin{array}{c} Y_{0}=\frac{{\text{OD}}_{1}-{\text{OD}}_{2}}{{\text{OD}}_{3}-{\text{OD}}_{4}}\times C_{0}÷C_{\text{prot}}. \end{array}$$

In this equation, *Y*_0_ represents AChE activity (*μ*mol/min/mg prot), OD_1_ represents the absorbance of the experiment group, OD_2_ represents the absorbance of the control group, OD_3_ represents the absorbance of the reference group, OD_4_ represents the absorbance of the blank group, *C*_0_ represents the standard concentration (1 mM), and *C*_prot_ represents the protein concentration of the sample.

### 2.5. Statistical Analyses

Nonselective and selective antifeeding ratios were calculated according to the following equations:

(4) $$\begin{array}{c} Y_{1}=\frac{S_{\text{ck}}-\text{S}}{S_{\text{ck}}}\times 100\%,\\ Y_{2}=\frac{S_{\text{ck}}-\text{S}}{S_{\text{ck}}+S}\times 100\%. \end{array}$$

In these equations, *Y*_1_ represents the nonselective antifeeding ratio (%), *Y*_2_ represents the selective antifeeding ratio (%), *S*_ck_ represents the average feeding area of the control group (mm), and *S* represents the average feeding area of the treated group (mm).

The nonselective and selective antifeeding ratios of larvae fed on diets containing different concentrations of ginsenosides were compared using SPSS Statistical 18.0. The effects of ginsenosides on the detoxification enzyme activity of * M. separata* larvae were also analyzed using SPSS Statistical 18.0. In all statistical tests, *P* values < 0.05 were considered statistically significant. All data are presented as the means ± SEM.

## 3. Results

### 3.1. Bioassay for Feeding Behavior

The nonselective antifeeding ratios of 4th-instar* M. separata* larvae at 8, 16, and 24 h are shown in Table 1. The total ginsenosides appeared to have significant antifeeding activity against* M. separata* larvae. The nonselective antifeeding ratios at different total ginsenoside concentrations were significantly different from those of the controls at 4, 8, and 12 h. As the total ginsenoside concentration increased, the nonselective antifeeding activity was enhanced. At 8 h, the antifeeding ratios of 2.0%, 1.0%, and 0.5% (MF) were 88.67%, 64.40%, and 47.36%, respectively.

The selective antifeeding ratios of the 4th-instar* M. separata* larvae are shown in Table 2. The 4th-instar* M. separata* larvae preferred to eat the leaves of the control; however, they did consume a small amount of the treated leaves. The total ginsenosides had significant inhibitory effect on* M. separata* larvae, and this effect increased with an increase in total ginsenoside concentration.

### 3.2. Enzymes Activity Tests

As the total ginsenoside concentration increased, the activity of GST also increased. GST activities first decreased and then increased with the passage of time. The inhibition ratio was the highest at 48 h, with 75.15%, 468.98%, and 31.86% at 2.0%, 1.0%, and 0.5% concentrations, respectively (see Figure 2).

The AChE activity of 4th-instar* M. separata* larvae was 79.0546 *μ*mol/min/mgprot before feeding on 2.0% (MF) total ginsenosides (see Figure 3); however, the AChE activities were depressed to 61.8998 (inhibition ratio, 21.70%), 31.5114 (inhibition ratio, 60.14%), and 35.0698 (inhibition ratio, 55.64%) *μ*mol/min/mgprot at 24 h, 48 h, and 72 h, respectively (see Figure 4). The ginsenosides inhibited the AChE activity of 4th-instar* M. separata* larvae, and the inhibition effect increased with the increase in ginsenoside concentration but did not decrease over time.

## 4. Discussion

Plant secondary metabolic substances are formed via secondary metabolic pathways and play indispensable roles in the evolution of plants. They have repellency, antifeeding, and toxic effects on phytophagous insects. For example, Momordicin I and II have significant antifeedant activity on second-instar larvae of* P. xylostella*, and the antifeeding rates were 80.39% and 74.09%, respectively [8]. Plant lectins also have repellency, antifeeding, and toxic effects on phytophagous insects such as caterpillars, tobacco hornworm, cotton leafworm, and beetles [29]. Azadirachtin causes mortality in* Nilaparvata lugens* (Stal) and destroys its ovarian follicle epithelial cells [30].

We compared the larval feeding behavior of * M. separata* on a diet containing different concentrations of total ginsenosides (MF: 2.0%, 1.0%, and 0.5%). The total ginsenosides imposed adverse effects on the feeding behavior of* M. separata* but to different extents (Tables 1 and 2). Comparison with control demonstrates that the consumption of leaves by* M. separata* was strongly affected by the total ginsenosides. If consumption is reduced, the energy required for physiological activities would also be reduced. Consequently, resistance is then reduced, and physiological metabolism would not be adequate.* M. separata* are sometimes found near cultivated* ginseng*; however, they rarely feed on the leaves of* ginseng*, which is the reason for choosing* M. separata* for this experiment.

**Figure 1:**
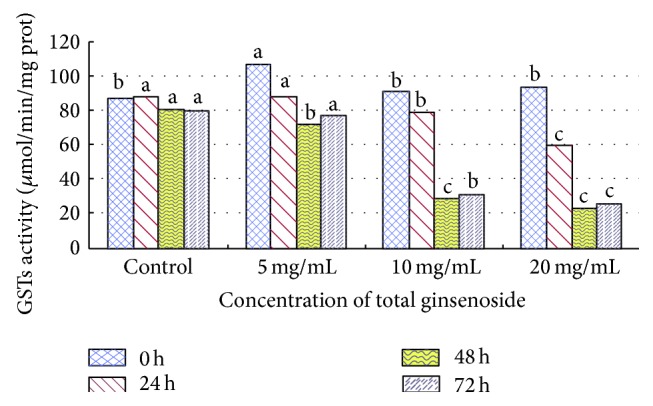
Effect of total ginsenosides on GST activity in 4th-instar * M. separata* larvae. Bars with different letters are significantly different from each other at *P* < 0.05 using the one-way ANOVA (SPSS 18.0). Data are means + SD.

**Figure 2:**
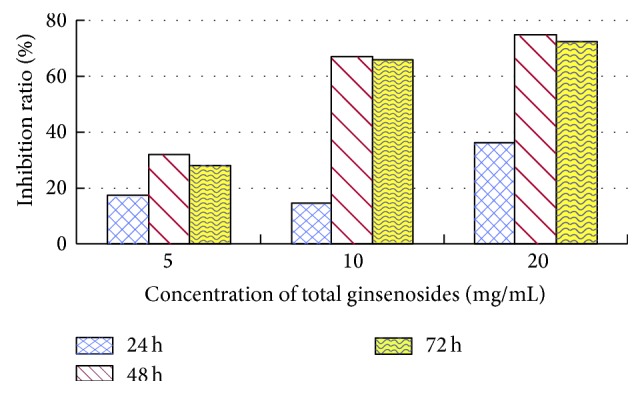
Inhibition ratio of total ginsenosides on GST activity in 4th-instar* M. separata* larvae.

**Figure 3:**
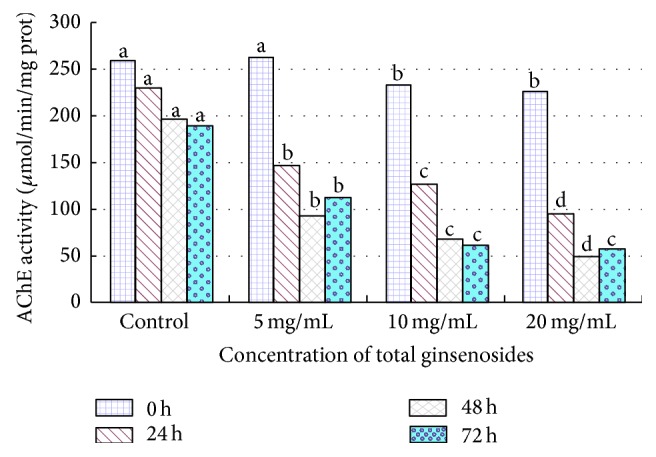
Effect of total ginsenosides on AChE activity in 4th-instar* M. separata* larvae. Bars with different letters are significantly different from each other at  *P* < 0.05 using a one-way ANOVA (SPSS 18.0). Data are means + SD.

**Figure 4:**
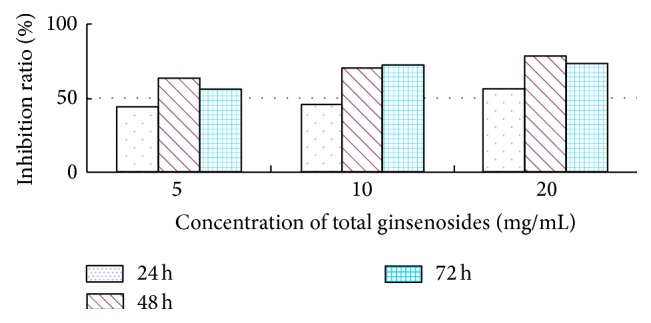
Inhibition ratio of total ginsenosides on AChE activity in 4th-instar* M. separata* larvae.

Total ginsenosides inhibited GST and AChE activities of* M. separata* larvae. If the GST activity was inhibited, the detoxification was reduced. The* M. separata* changed feeding behaviors and fed on the grain sorghum leaves that were treated with total ginsenosides. In this way, they could reduce the damage from outsiders and protect themselves. It is the same for AChE; their neurotransmission slows down when AChE activity is inhibited and* M. separata* did feed on a small amount. The total ginsenosides had antifeeding activity against* M. separata* larvae and inhibitory effects on the activities of GST and AChE. Glutathione S-transferase (GST) and acetylcholinesterase (AChE) play important roles in insect metabolism and resistance to insecticides [31]. This study demonstrates that after treating* M. separata* with total ginsenosides GST activities decreased. This indicates that total ginsenosides have no toxic effects in vivo and activates the defense system of insects but could also inhibit GST activity. GSTs widely exist in insects and catalyze the nucleophilic reaction of glutathione (GSH) with a variety of electrophilic compounds. GSTs play an important role in exogenous substance biotransformation, drug metabolism, and protection of the organism against oxidative damage [32]. GST activity was inhibited by total ginsenosides, and detoxification was reduced in 4th-instar* M. separata* larvae (see Figure 1). Thus, they suffered damage from toxic substances and these substances are harmful to living larvae.

AChE is an important neurotransmitter enzyme mainly distributed in the brain and central nervous system [33]. AChE is localized at the surface of nerve cells and can decompose neurotransmitter acetylcholine, which is released from the nerve endings and guarantees normal nerve conduction [34]. If its activity is inhibited, the acetylcholine released in the synaptic gap cannot be degraded, and insects will have toxicity symptoms and possibly mortality [35]. Several essential oils from aromatic plants, monoterpenes, and natural products have all been shown to be inhibitors of AChE [36]. Pulegone-1,2-epoxide, isolated from a Verbenaceae medicinal plant (*Lippia stoechadifolia* L. (Poleo)), showed an irreversible inhibition of the AChE in house fly and Madagascar roach [37]. This experiment indicates that the total ginsenosides inhibit AChE activity directly. On the basis of the research mentioned earlier and the comprehensive analysis of our findings, we conclude that the decline of AChE activity in* M. separata* larvae indirectly indicates damage to nerve cells, which induce AChE photoinactivation and death caused by the disruption of normal nerve conduction. Total ginsenosides have inhibitory allelopathic effects on* M. separata*, which were beneficial for researching the allelopathic potential of ginsenosides.

## Figures and Tables

**Table 1:** Nonselective antifeeding ratio of different total ginsenoside concentrations on 4th-instar *M. *separata larvae.

Concentration of total ginsenosides (%)	8 h		16 h		24 h	
	Average feeding area (mm^2^)	Nonselective antifeeding ratio (%)	Average feeding area (mm^2^)	Nonselective antifeeding ratio (%)	Average feeding area (mm^2^)	Nonselective antifeeding ratio (%)
Control	1664.67 ± 107.04 a	—	1770.00 ± 0.00 a	—	1770.00 ± 0.00 a	—
0.5	876.33 ± 333.10 b	47.36	1529.67 ± 28.02 a	13.58	1440.67 ± 89.80 a	18.61
1.0	592.67 ± 224.13 b	64.40	1524.00 ± 75.29 a	13.90	1408.00 ± 201.27 a	20.45
2.0	188.67 ± 81.59 c	88.67	757.67 ± 453.24 b	57.19	483.33 ± 358.60 b	72.69

Data are presented as the means ± SE. Means in the same column followed by different letters are significantly different at the level *P* < 0.05. Prior to analysis of variance (SPSS 18.0), the homogeneity of variance was tested in each statistic test.

**Table 2:** Selective antifeeding ratio of different total ginsenoside concentrations on 4th-instar *M.* separata larvae.

Concentration of total ginsenosides (%)	8 h		16 h		24 h	
	Average feeding area (mm^2^)	Selective antifeeding ratio (%)	Average feeding area (mm^2^)	Selective antifeeding ratio (%)	Average feeding area (mm^2^)	Selective antifeeding ratio (%)
0.5	638.33 ± 34.53	34.19	450.33 ± 40.45	36.62	511.33 ± 9.61	23.28
Control	1301.67 ± 77.02	—	970.67 ± 52.78	—	821.67 ± 39.00	—
1.0	514.67 ± 80.75	44.29	470.67 ± 24.50	32.00	527.33 ± 25.74	29.23
Control	1332.33 ± 51.50	—	913.67 ± 33.47	—	963.00 ± 36.10	—
2.0	267.00 ± 95.79	62.49	285.33 ± 23.46	46.18	391.00 ± 22.37	42.46
Control	1156.67 ± 127.38	—	775.00 ± 50.48	—	968.00 ± 24.06	—

Data are presented as the means ± SE. Homogeneity of variance was tested in each statistic test.
